# Ionized Plasma Jet Therapy for the Treatment of Striae Distensae

**DOI:** 10.7759/cureus.115051

**Published:** 2026-08-23

**Authors:** Manjiri Oke, Sanika R Patil, Milind Borkar

**Affiliations:** 1 Department of Dermatology, NKP Salve Institute of Medical Sciences and Research Centre and Lata Mangeshkar Hospital, Nagpur, IND; 2 Department of Dermatology, Jannayak Birsa Munda Government Medical College, Nandurbar, IND

**Keywords:** ionized plasma therapy, ipjt, jet plasma therapy, stretch marks, striae distensae

## Abstract

Introduction: Striae distensae are common linear atrophic skin lesions caused by alterations in collagen, elastin, and other components of the extracellular matrix. Available treatments often provide inconsistent improvement, particularly in mature striae albae.

Objective: This study aimed to evaluate the safety and efficacy of ionized plasma jet therapy (IPJT) in the treatment of striae distensae.

Methodology: This open-label experimental study included 30 patients aged 15-49 years who met the predefined eligibility criteria. Participants underwent four sessions of IPJT using the Jett Plasma Lift Medical device (JETT; COMPEX, spol. s r.o., Brno, Czech Republic). Ablative and non-ablative treatments were administered alternately at 15-day intervals, with two sessions of each modality. Clinical improvement was evaluated through blinded photographic assessment using a five-point scale ranging from no improvement to excellent improvement. Procedural pain and adverse effects, including edema, itching, erythema, hypopigmentation, and hyperpigmentation, were assessed during follow-up visits for up to 90 days.

Results: The mean age of the participants was 28 ± 5.4 years; 18 (60.0%) were female and 12 (40.0%) were male. Striae albae were present in 28 (93.3%) patients, whereas 2 (6.7%) had striae rubrae. Excellent improvement was observed in 5 (16.7%) patients, good improvement in 19 (63.3%), and moderate improvement in 6 (20.0%). Post-inflammatory hyperpigmentation occurred in three (10.0%) patients and resolved following treatment with a topical depigmenting agent. The mean procedural pain score was 2.2 ± 0.6 on the visual analogue scale. No persistent edema, erythema, itching, scarring, hypopigmentation, or serious treatment-related adverse events were observed.

Conclusion: IPJT demonstrated promising clinical improvement, with mild procedural discomfort and a low frequency of temporary adverse effects in patients with striae distensae. However, larger randomized controlled trials using validated assessment tools and balanced representation of different striae subtypes are required to further establish its safety and efficacy.

## Introduction

Stretch marks, also known as striae distensae (SD), are well-defined, atrophic, linear cutaneous lesions that develop as a result of alterations in connective tissue associated with growth spurts, obesity, or corticosteroid use. Although the precise mechanisms underlying the pathophysiology of SD remain unclear, several researchers have suggested that abnormalities in collagen fibrils, elastic fibers, or other components of the extracellular matrix (ECM) may contribute to their development [1]. Disruption of the elastin fiber network alters the viscoelastic properties of the affected skin, making it less firm, elastic, and deformable than normal skin [2]. Stretch marks exhibit a characteristic maturation process, initially presenting as striae rubrae and subsequently progressing to striae albae. Striae rubrae are typically erythematous and may occasionally be edematous, whereas striae albae are hypopigmented, depressed, atrophic lesions with a wrinkled appearance. Although generally asymptomatic, stretch marks can be cosmetically distressing [3]. Early lesions, or striae rubrae, generally respond better to treatment than older, more established striae albae. Despite the availability of various treatment modalities, the management of stretch marks has historically yielded unsatisfactory results and remains challenging for both patients and clinicians [4]. Topical therapies have demonstrated limited efficacy. More recently, ionized plasma therapy has emerged as a potential treatment modality for SD. Plasma is considered the fourth state of matter and can be generated by heating a gas or subjecting it to a strong electric field [5]. In plasma generators, ionization of atmospheric gases produces a concentrated microplasma beam, and the resulting energy is applied to the targeted areas for therapeutic purposes [6].

This study evaluated the safety and efficacy of ionized plasma jet therapy (IPJT) in the treatment of SD.

## Materials and methods

This was an open-label experimental study involving patients with SD who presented to the dermatology outpatient department of a tertiary care center. The study was approved by the Institutional Ethics Committee before commencement. The inclusion and exclusion criteria are presented in Table 1.

**Table 1:** Inclusion and exclusion criteria

Inclusion criteria	Exclusion criteria
Patients aged 15-49 years	Patients with a tendency toward keloid formation
Patients diagnosed with striae distensae	Pregnant or lactating patients
Patients with no significant medical history	Patients with an active infection at or near the treatment site
Patients who had not received any surgical or non-surgical treatment for striae distensae during the preceding six months	Patients with bleeding disorders
Patients willing to undergo ionized plasma jet therapy according to the study protocol	Patients receiving ongoing anticoagulant therapy
Patients who provided written informed consent	Patients with implanted medical devices
Participants younger than 18 years who provided written assent along with written consent from a parent or legally authorized representative	Patients with pacemakers

The study protocol was approved by the Institutional Review Board. Written informed consent was obtained from participants aged 18 years or older. Participants younger than 18 years provided written assent along with written informed consent from a parent or legally authorized representative before undergoing IPJT. Eligible patients were treated using the JETT PLASMA LIFT MEDICAL device (JETT; COMPEX, spol. s r.o., Brno, Czech Republic).

Topical anesthesia was applied to the affected area and left in place for 45 minutes. The treatment area was then cleansed with povidone-iodine (Betadine) and normal saline. During the first treatment session, an ablative probe of the IPJT device was used at an intensity of 5-6 arbitrary device units. The borders of the striae were ablated using intermittent dot application, while the central portion of the striae was treated using a brush technique. The patient was reassessed after 15 days. During the second treatment session, the striae were treated using a non-ablative probe at an intensity of 1-5 arbitrary device units for 20 minutes. The ablative and non-ablative procedures were subsequently repeated alternately at 15-day intervals. A total of four treatment sessions, two ablative and two non-ablative, were performed for each patient. A topical antibiotic was prescribed after each procedure. Crust formation resolved spontaneously within seven days. Patients were followed up at 7, 15, 30, 45, and 90 days after treatment. During follow-up, patients were evaluated for edema, pain, itching, and local hyperemia, as well as for the development of hyperpigmentation or hypopigmentation in the treated area. Pain was assessed using a 100-mm Visual Analog Scale (VAS). At baseline, the size and number of lesions were recorded by a blinded evaluator. Clinical improvement was assessed by comparing standardized pre- and post-treatment photographs using a previously published five-point quartile grading scale [4]: 0, no improvement; 1, mild improvement (1%-25%); 2, moderate improvement (26%-50%); 3, good improvement (51%-75%); and 4, excellent improvement (76%-100%).

Clinical assessment was performed using photographic evaluation based on the five-point grading scale described in Table 2. 

**Table 2:** Five-point scale used for clinical assessment

Pointer grades	Symptoms resolution
0	No improvement
1	Mild (0%-25%)
2	Moderate (26%-50%)
3	Good (51%-75%)
4	Excellent (76%-100%)

## Results

The study included 30 patients, with a mean age of 28 ± 5.4 years. Females constituted 18 (60.0%) participants, while 12 (40.0%) were males. Fitzpatrick skin type IV was present in 21 (70.0%) patients and type III in 9 (30.0%). Most participants had striae alba, accounting for 28 (93.3%) cases, whereas two (6.6%) had striae rubrae. Following IPJT, no patient showed grade 0 and grade 1 improvement, while grade 2, grade 3, and grade 4 improvements were reported in 6 (20.0%), 19 (63.3%), and 5 (16.6%) patients, respectively (Table 3). 

**Table 3:** Patient profile of the study participants

Parameter	Mean ± standard deviation/number (frequency %)
Age	28 ± 5.4
Male	12 (40)
Females	18 (60)
Fitzpatrick skin type 03	9 (30)
Fitzpatrick skin type 04	21 (70)
Striae alba	28 (93.3)
Striae rubra	2 (6.6)
Improvement pointer grades (0)	0 (0)
Improvement pointer grades (1)	0 (0)
Improvement pointer grades (2)	6 (20.0)
Improvement pointer grades (3)	19 (63.3)
Improvement pointer grades (4)	5 (16.6)
Hyperpigmentation	3 (10)

The abdomen was the most commonly affected site, observed in 12 (40.0%) patients, followed by the thighs in 8 (26.7%) and the shoulders in 7 (23.3%). The back was the least frequently affected site, observed in 3 (10.0%) patients (Table 4).

**Table 4:** Site of striae on the body

Site	Number (percentage)
Shoulder	7 (23.3)
Back	3 (10)
Abdomen	12 (40)
Thigh	8 (26.6)

**Figure 1:**
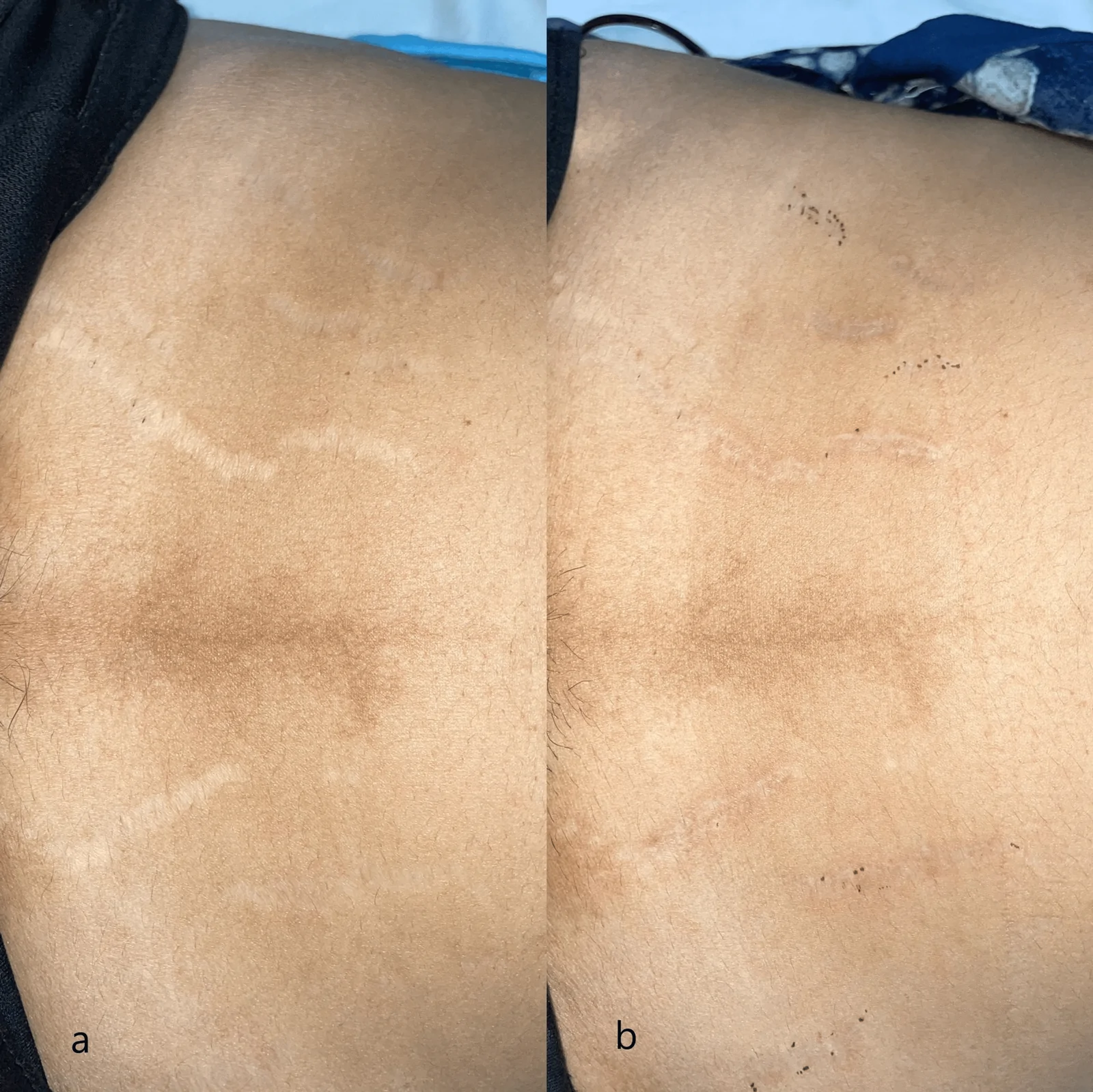
Excellent clinical response to IPJT for SD over the lower abdomen. (a) Pretreatment appearance on day 0. (b) Post-treatment appearance on day 90 IPJT: ionized plasma jet therapy. SD: striae distensae.

Excellent improvement in the appearance of the striae was observed in five patients (16.6%) (Figure 1).

**Figure 2:**
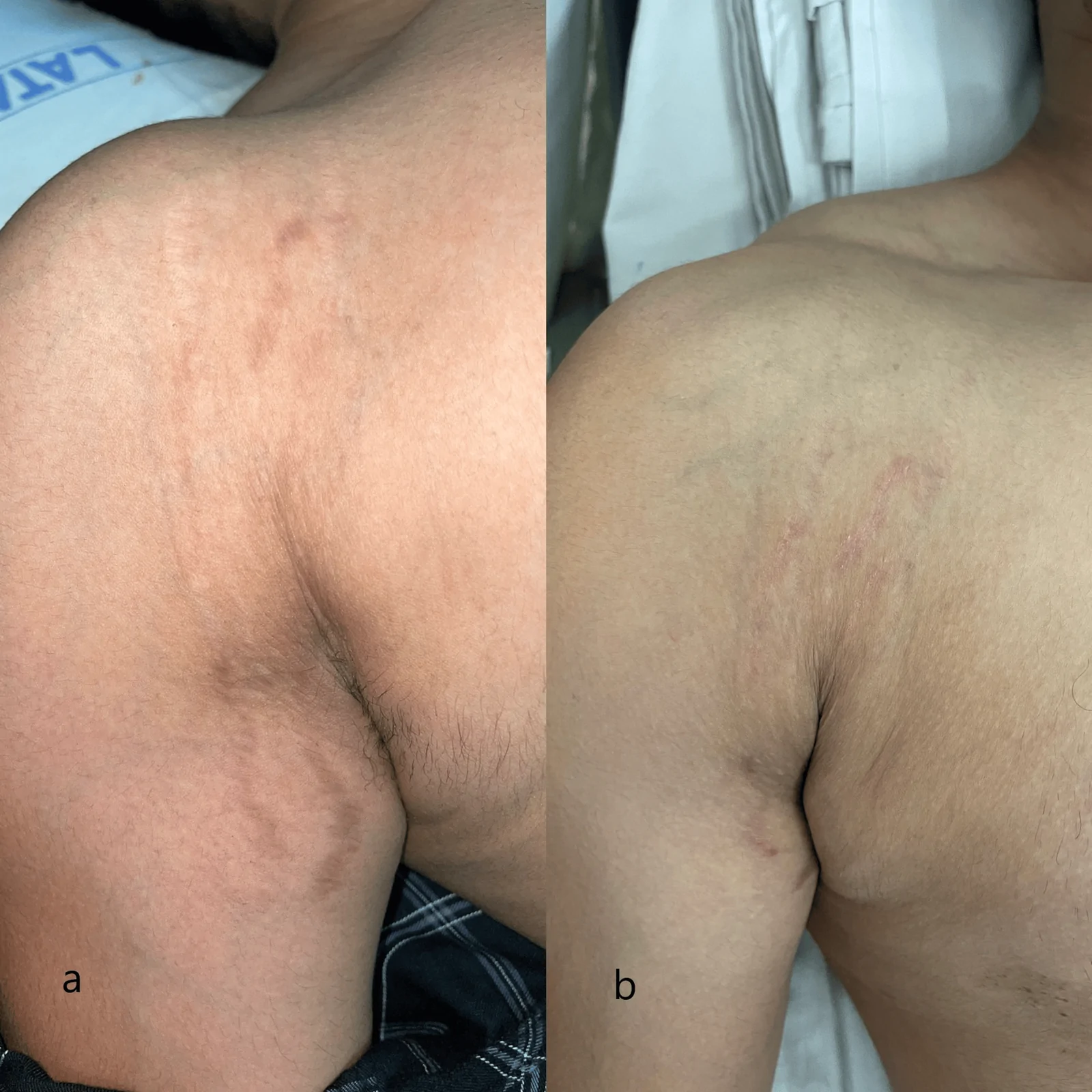
Good clinical response of striae distensae (SD) over the shoulder. (a) Pretreatment appearance on day 0. (b) Post-treatment appearance on day 90 SD: striae distensae.

**Figure 3:**
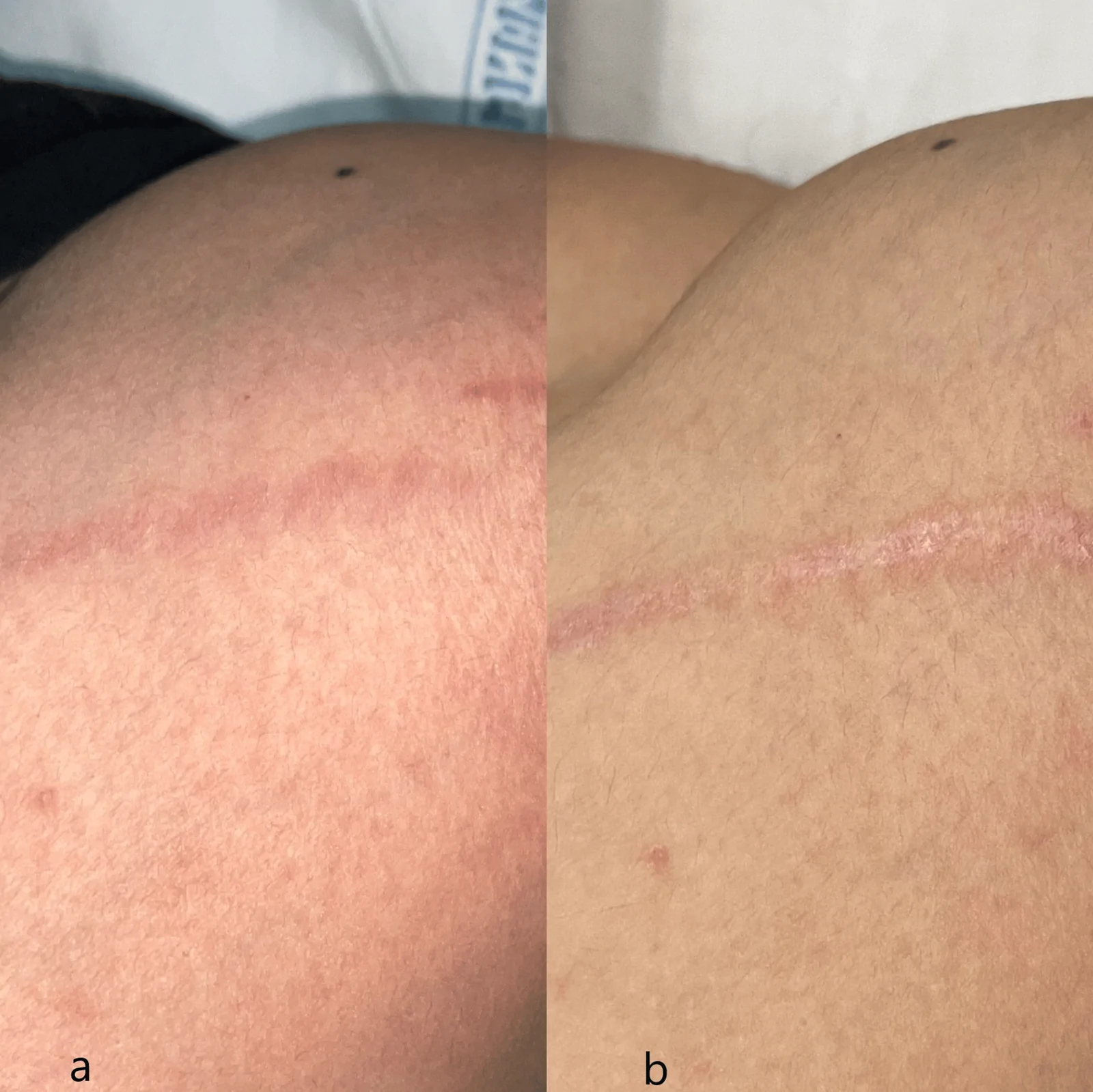
Moderate clinical response of SD over the shoulder. (a) Pretreatment appearance on day 0. (b) Posttreatment appearance on day 90 SD: striae distensae.

Good and moderate improvement was observed in 19 patients (63.3%) and 6 patients (20.0%), respectively (Figures 2, 3).

**Figure 4:**
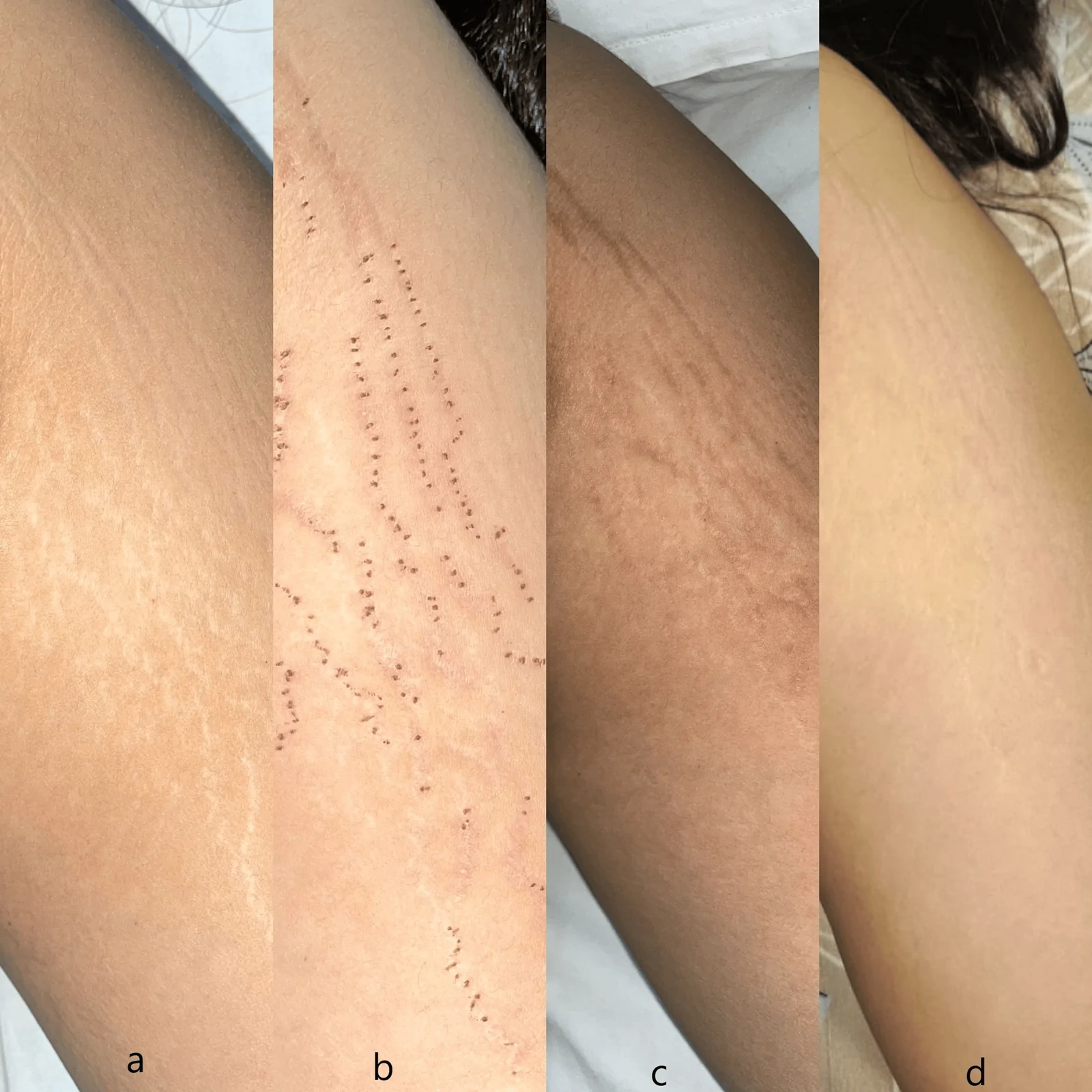
Progression of treatment in SD. (a) Pretreatment appearance on day 0. (b) Post-ablative IPJT appearance on day 30. (c) Post-non-ablative IPJT appearance on day 45, showing hyperpigmentation. (d) Appearance on day 90 following treatment with a topical depigmenting cream SD: striae distensae, IPJT: ionized plasma jet therapy.

Hyperpigmentation was observed in three patients and was successfully treated with a topical depigmenting agent (Kojivit Plus Gel) (Figure 4).

## Discussion

The present study evaluated IPJT as a treatment for SD and demonstrated encouraging short-term clinical improvement with relatively few adverse effects. Among the 30 treated patients, good improvement was observed in 19 (63.3%), excellent improvement in 5 (16.7%), and moderate improvement in 6 (20.0%). Post-inflammatory hyperpigmentation occurred in three (10.0%) patients and resolved with topical treatment. No persistent erythema, edema, itching, hypopigmentation, scarring, or serious treatment-related complications were observed. These findings suggest that IPJT may provide clinically visible improvement while maintaining an acceptable short-term safety profile.

Several treatment modalities have been investigated for the management of SD [6-15]. The 1064-nm long-pulsed Nd laser has demonstrated clinical improvement in striae rubrae because of its affinity for vascular targets within these lesions. The improvement in erythema is attributed to the absorption of laser energy by oxyhemoglobin. In addition, long-pulsed Nd laser treatment, similar to other light-based modalities, may stimulate neocollagenesis, thereby reducing dermal atrophy and improving the appearance of immature striae [13,15]. Other modalities have also shown varying degrees of efficacy. Focused phototherapy using UVB/UVA sources has been reported to improve hypopigmentation in striae alba, although evidence in this area remains limited [6,9]. The use of V-EMF, involving electromagnetic fields and negative pressure, has been associated with improvement in stretch-mark coloration, an increased number of melanocytes, enhanced vascularization, and epithelial thickening [7,10]. Fractional ablative CO2 laser therapy is another relatively safe and effective treatment option for SD. The addition of platelet-rich plasma (PRP) has been reported to provide marginal additional efficacy; however, hyperpigmentation remains an important adverse effect that requires consideration and management [8,11].

Striae rubrae are a prominent source of cosmetic concern and anxiety, particularly among adolescents. In comparative studies, a combination of microdermabrasion, salicylic acid, and retinol yellow peel produced better outcomes than several alternative treatment modalities. Topical tretinoin showed no improvement, whereas Nd laser therapy, mesotherapy, and microdermabrasion combined with trichloroacetic acid (MDA + TCA) produced mild-to-moderate improvement [12,14].

Plasma, the fourth state of matter, is generated when a neutral gas undergoes ionization. When sufficient energy is applied to a gas by a radiofrequency generator, electrons can be separated from their atoms, resulting in ionization and the formation of charged particles. In IPJT, the energy generated by the ionization of ambient gas between the device and the skin is utilized for treatment. In the non-ablative mode, a silver applicator is used at intensities ranging from 1 to 5. The spark discharge generates a flow of negative electrons and positive ions. The discharge is produced by direct current, with a negative charge at the device tip. Application of voltage alters the potential of cell membranes, increases the permeability of intercellular channels, and modifies ion distribution, resulting in swelling of the targeted tissue. The device consists of a handpiece with a sterile tip [16]. The resulting plasma spark may coagulate dermal vessels and sublimate the outermost layers of the skin while avoiding severe thermal damage. These properties may contribute to the clinical effects observed with IPJT. Additional advantages of the procedure include a favorable safety profile, minimal downtime, and ease of use. IPJT devices are suitable for office-based procedures because they are compact, lightweight, and relatively simple to operate [17].

Several limitations of this study should be noted. First, the study lacked a control arm. In the absence of a controlled comparator, the observed photographic improvements cannot be definitively attributed to IPJT, particularly because spontaneous or partial resolution of striae rubrae over a 90-day period has been documented. Second, the sample had an uneven distribution of striae subtypes. Of the 30 enrolled patients, the vast majority (n = 28) presented with striae alba, whereas only two presented with striae rubrae. Because striae alba is generally considered more resistant to treatment, this imbalance limits the generalizability of the findings. Consequently, although the safety profile of IPJT was favorable, definitive conclusions regarding its overall efficacy across all types of SD cannot be drawn from these data alone. Future randomized controlled trials involving larger and more evenly distributed cohorts are required to establish the clinical efficacy of IPJT.

The methodology used to assess clinical improvement also presents certain limitations. The five-point photographic scale used in this study was an in-house instrument and has not been formally validated against published outcome measures for SD. Although sufficient intra-observer consistency was observed, formal inter-rater reliability was not assessed. Future studies should prioritize validated SD-specific scoring systems and the use of independent, blinded assessors to facilitate a more objective evaluation of treatment response. Finally, the relatively small sample size of the adolescent subgroup (n = 5) represents an additional limitation. Although no specific safety concerns related to IPJT emerged in this age group, the study was not sufficiently powered to detect rare adverse events or subtle differences in neocollagenesis specifically among patients aged 15-17 years. Future studies involving dedicated pediatric cohorts are warranted to confirm the efficacy and safety of IPJT in this population.

## Conclusions

IPJT demonstrated promising short-term clinical improvement in patients with SD, with most participants achieving good or excellent improvement and only mild, temporary adverse effects. However, the absence of a control group, small sample size, predominance of striae alba, use of a non-validated assessment scale, and limited follow-up preclude definitive conclusions regarding treatment efficacy. These findings should therefore be considered preliminary. Larger randomized controlled trials incorporating validated outcome measures, blinded assessment, balanced representation of striae subtypes, and longer follow-up are warranted to establish the safety, efficacy, and durability of IPJT for SD.
